# Ethnomedicinal and Phytopharmaceutical Properties of *Syzygium samarangense* (Blume) Merr. & L.M. Perry: A Gateway to Future Drug Development

**DOI:** 10.1002/cbdv.202502295

**Published:** 2026-10-22

**Authors:** Sujit Kumar Patro, Rudra Narayan Sahoo, Ashirbad Nanda, Shasank S. Swain

**Affiliations:** ^1^ School of Pharmacy and Life Sciences Centurion University of Technology and Management Bhubaneswar Odisha India; ^2^ Department of Pharmacognosy Roland Institute of Pharmaceutical Sciences Berhampur Odisha India; ^3^ School of Pharmaceutical Sciences Department of Pharmacology Siksha ‘O’ Anusandhan Deemed to be University Bhubaneswar India; ^4^ School of Biotechnology Centurion University of Technology and Management Bhubaneswar Odisha India; ^5^ Research and Development Division Salixiras Research Private Limited Bhubaneswar Odisha India

**Keywords:** medical plants, *Syzygium samarangense*, ethnomedicinal values, biological activities, plant‐derived bioactive molecules

## Abstract

Recently, natural products and plant‐based regimens are widely recognized as potential complementary and alternative therapeutic solutions. Traditionally, ethnomedicinal knowledge, validated through scientific evidence, has been a basis for modern drug discovery, emphasizing the importance of updated phytopharmacological knowledge of medicinal plants. *Syzygium samarangense* (Blume) Merr. & L.M. Perry, commonly known as wax apple, is a tropical plant belonging to the family Myrtaceae and is rich in diverse phytochemicals and bioactive compounds that contribute to its medicinal properties. Traditionally, *S. samarangense* leaves, bark, fruit, and seeds are used in ethnomedicine for treating diabetes, infection, inflammation, and gastrointestinal disorders. Phytochemical studies have shown the presence of some bioactive constituents such as flavonoids (quercetin and myricetin), tannins, terpenoids (betulinic acid and thymol), and phenolic acids (gallic acid and ferulic acid) with promising pharmacological activities and high translational potential due to their well‐established therapeutic properties. However, its successful integration in the form of a natural remedy or individual therapeutic candidate in modern pharmacotherapy needs to address the challenges of bioavailability, long‐term safety, standardization of formulations, and elucidation of precise molecular mechanisms, despite its pharmacological promise. In conclusion, the present review updates the ethnomedicinal, phytochemical, and pharmacological importance of *S. samarangense* and provides useful information to academic and pharmaceutical research to identify new drug candidates.

## 1 Introduction

Ethnomedicine is the traditional medicine of the various cultures of the world. These practices depend on the use of locally available plants, minerals, and animal products for the treatment of different ailments, especially in areas where access to modern medical facilities is limited [1, 2, 3, 4]. Thus, almost 80% of the heritage communities of the hillside use different extracts and formulations of medicinal plants in their day‐to‐day lives, especially in the higher Asian and African communities, which are highly used [2, 5, 6, 7]. Different regions and countries recognize ethnomedicines under different names, including traditional Chinese medicine (TCM), Indian Ayurveda, African traditional medicine, Amazonian ethnomedicine, and Native American medicine [1, 8, 9, 10]. Currently, researchers scientifically investigate these ethnomedicines on various experimental platforms for their potential application in mainstream medicine [11, 12]. Thus, ethnomedicine offers important clues for contemporary medical investigation and emphasizes the significance of cultural diversity and sustainability in health care practices. Both aromatic and medicinal plants are some of the most crucial and conservative ethnomedicinal sources around the globe [12, 13]. Natural products or phytochemicals have proven their worth in modern medicine by providing a variety of drug molecules like quinine, Taxol, vincristine, vinblastine, caffeine, morphine, digoxin, atropine, and aspirin [12, 14].

Traditional knowledge on natural products can form a basis for discovery of new drugs and management of affordable healthcare [12, 13]. The application of ethnomedicine to modern medicine encourages holistic and patient‐oriented health care and promotes research and scientific innovations. It has positive externalities on the local economy and resilience to health shocks [15, 16]. Conservation of ethnomedicinal practices is important for the conservation of diverse and sustainable health care resources and for cultural continuity for future generations [15, 16]. Therefore, knowledge about the chemical diversity of bioactive metabolites is a prerequisite for the discovery of new drugs [17]. The chemical diversity of plant metabolites is also critical for biodiversity and ecosystem health; it enables sustainable use of natural resources that are critical for medical, environmental, and economic resilience for future generations [15, 16, 17].

Ethnomedical knowledge is generally accepted from the primitive era, but it is often not backed up by empirical evidence from rigorous scientific experimentation [18, 19]. The Indian subcontinent and Asia in general are rich in botanical diversity and have a long tradition of medicinal practices that are deeply rooted in their culture [20, 21, 22]. For translational purposes, it is necessary to focus our research on specific diseases and disorders to bridge the gap between the traditional ethnomedicinal knowledge and modern scientific insights [16, 23]. Currently, there is a growing trend to explore the therapeutic effectiveness of traditional plant parts, especially to reduce antibiotic consumption, medical costs, drug unavailability, and drug resistance by using active natural plant regimens for a sustainable society [24, 25, 26]. A diverse range of parties, such as academic institutions, research organizations, governments, nongovernmental organizations, international agencies, and the pharmaceutical industry, are involved in the promotion and improvement of ethnomedicine and natural‐derived products [13, 19, 26, 27]. The promotion and enhancement of research, conservation, and education are important to tap the potential of traditional knowledge and natural resources for global health care and biodiversity conservation [15, 16, 17].

This review is about *Syzygium samarangense* (Blume). Merr. & L.M. Perry from the Myrtaceae family, a widely edible and medicinal plant from Asia. It looks at its chemical diversity, biological potency, and phytopharmaceutical properties. Through an extensive literature search, we collected its longest‐standing ethnomedicinal uses and scientifically investigated biological activities with isolated bioactive metabolites from different plant parts. Here we also highlighted the challenges and future of reported bioactive metabolites as drug molecules in mainstream medicine. In short, preserving traditional knowledge and updated scientific reports always plays a crucial role in the utilization of natural regimens in modern drug discovery towards fulfilment of drug unavailability.

## 2 Geographical Distribution and Diversity of *S. samarangense*

### 2.1 Botanical and Geographical Description of *S. samarangense*

*S. samarangense* (Blume) Global. Merr. & L.M. Perry, also known as wax apple, Java apple, or water apple, is a tropical tree species belonging to the family Myrtaceae [28, 29, 30]. It is indigenous to the Malay Peninsula, Andaman and Nicobar Islands, and the Greater Sunda Islands and is now widely cultivated in the tropics and subtropics [29, 30]. The adaptability and agricultural relevance of *S. samarangense* can be explored from the morphological, genetic, and ecological perspectives (Figure 1). The species exhibits considerable morphological variation, especially in fruit characters [28, 30]. Fruits are bell‐shaped, pear‐shaped, or round in shape. The size ranges from 3 to 10 cm long. Fruits present a wide range of colors, including white, green, pink, red, and purple to black [28, 30, 31]. Texture is crisp or spongy, and flavor ranges from bland to sweet, with some cultivars having a strong aroma. Such differences are due to genetic factors, environmental conditions, and the practices of selective breeding [29, 30].

**FIGURE 1:**
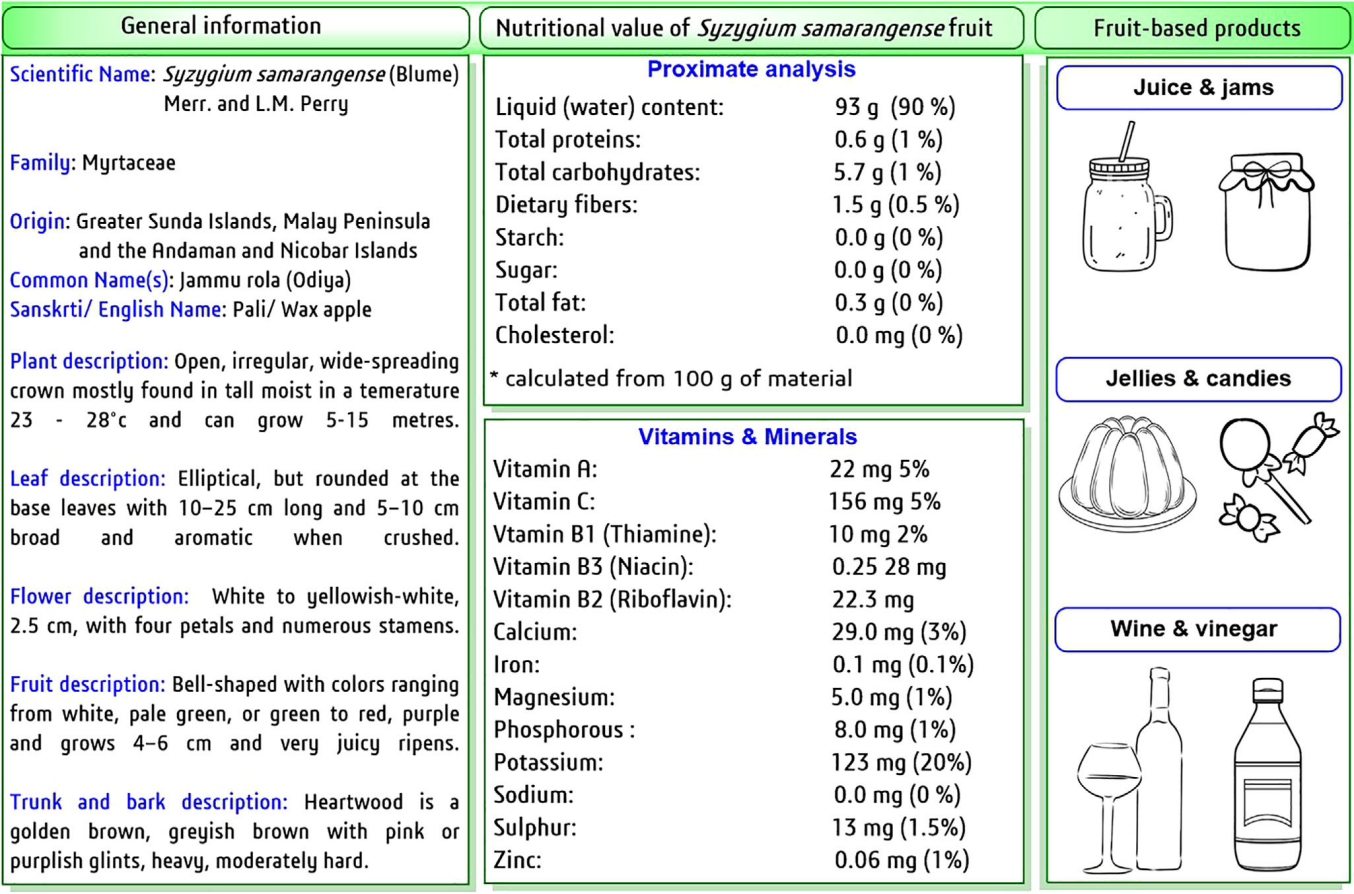
Botanical and diversity of *S. samarangense* along with nutritional values of their fruits as per report. These information's was collected from extensive literature search.

Genetic diversity of *S. samarangense* with different genotypes was found; many of them were selected or bred for specific traits, such as disease resistance, better fruit quality, and increased yield [29, 30, 32]. Knowledge of the genetic basis of these traits is important for breeding programs aimed at improved agronomic and nutritional traits in elite cultivars. *S. samarangense* is widely distributed across diverse habitats, ranging from coastal lowlands to inland regions at varying altitudes, and can adapt to different soil conditions [30, 31]. It prefers loamy, well‐drained soils rich in organic matter but can also grow in sandy or clayey soils. It is tolerant to environmental fluctuations and thus would be a viable candidate for cultivation in different agroclimatic zones. *S. samarangense* has variable morphological characters, genetic composition, and ecological adaptability of agricultural and economic importance [30, 31]. This diversity should be preserved to enable the sustainable cultivation and utilization of this valuable tropical fruit species to continue its role in food security, horticultural innovation, and medicinal applications.

### 2.2 Nutritional Values and Fruit‐Based Products From *S. samarangense*

The fresh fruit of *S. samarangense* is primarily composed of water, which accounts for approximately 90% of its composition. It also contains a small amount of protein, carbohydrates, and dietary fiber, and it contains a negligible amount of fat and cholesterol. As a result, it is a fruit that is low in calories and hydrating [26, 27, 28, 29, 30]. It is an excellent source of a wide range of vitamins, including vitamins A, C, B1, B2, and B3; minerals (potassium in particular, as well as calcium, magnesium, phosphorus, sulfur, and trace amounts of iron and zinc) Figure 2 [26, 27, 28, 29, 30]; and various other nutrients. As a result of the fruit's subsequent processing into value‐added products like juices and jams, jellies and candies, and fermented beverages like wine and vinegar, the fruit's economic significance to the food industry is further highlighted [26, 27, 28, 29, 30].

**FIGURE 2:**
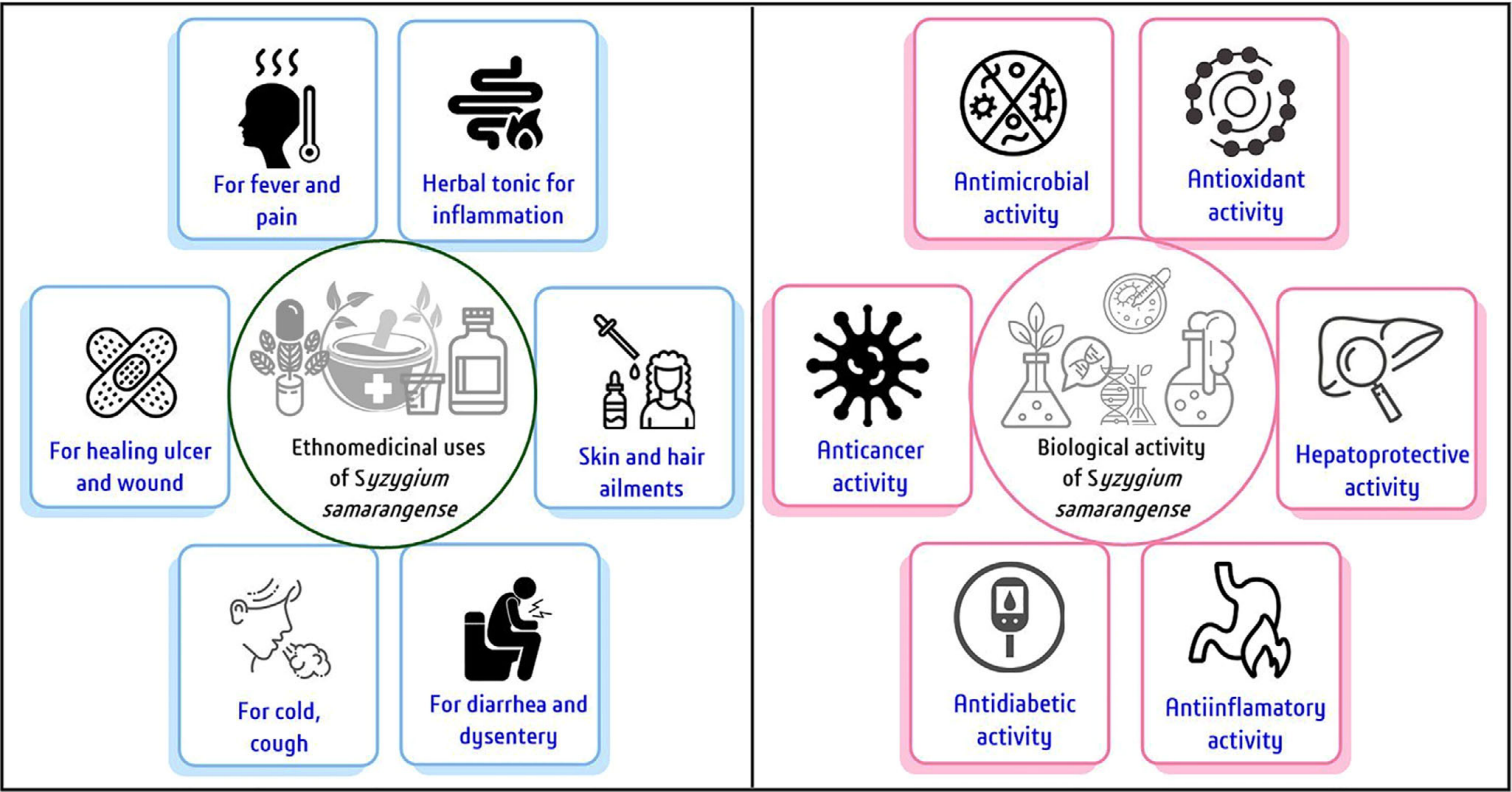
Graphical presentation of ethnomedicinal uses by ethnic community and explored biological activities of *S. samarangense* plant extracts by different research groups.

## 3 Medicinal Properties of *S. samarangense*

### 3.1 Traditional and Ethnomedicinal Values of *S. samarangense*

The genus *Syzygium* (Myrtaceae) comprises almost 500 species, cultivated especially in tropical and subtropical regions around the world. Among all species, *S. samarangense* is one of the most widely distributed and well‐known for its ethnomedicinal properties [29, 33, 34]. People have traditionally used nearly all parts of *S. samarangense*, including the roots, leaves, and flowers, to cure various ailments such as bronchitis, asthma, lower blood sugar, fever, pathogenic infections, gut spasms, and renal diseases (Figure 2). Mostly, people value the wax apple for its high nutritional content, as the fruit is rich in vitamins, minerals, and antioxidants [29, 35]. Traditionally, it is consumed for its fiber content to aid in digestion, and it is mildly laxative. It is also a source of hydration because of its high water content, especially in hot climates [29]. The leaves of *S. samarangense* are used in traditional medicine as a tea infusion to relieve stomachs and aid digestion. Leaf extracts are used in traditional medicine for diabetes management [29, 36]. Studies suggest that compounds in the leaves may help lower blood sugar levels. Furthermore, leaf extracts have been used as antiseptics and for treatment of infections, inflammation, and associated conditions such as arthritis and rheumatism [29, 36]. Flowers of the plant are believed to have anti‐inflammatory effects in traditional medicine and some studies; thus, they are used in poultices to reduce pain and swelling [29, 36]. Traditionally bark decoctions are used for their astringent action in the treatment of diarrhoea and dysentery. Furthermore, because of its antimicrobial properties, bark is used on wounds and ulcers for healing and preventing infections [29, 35, 37]. Traditional practitioners utilize seed extracts for the treatment of diarrhoea and gastrointestinal disturbances and to reduce oxidative stress (Figure 2). The roots of the wax apple tree are also used in traditional herbal medicine for many skin conditions and for general skin health improvement.

### 3.2 Biological Activities of Values of *S. samarangense*

To date, many studies have used different parts of the plant to test the biological activities to support the traditional medical claims about *S. samarangense* [34, 35, 36, 38]. The leaf extract has shown a pronounced hypoglycaemic effect in diabetic rat models mainly due to its flavonoid and saponins content, and therefore, it could be a potential natural antidiabetic agent [38, 39, 40]. Moreover, leaf and bark extracts have been found to show promising antimicrobial activity against bacterial and fungal strains [36, 41, 42]. Among the different plant parts, leaf extracts have shown the highest antioxidant capacity, which is linked to their presence of a rich amount of phenolic and flavonoid constituents [26, 30]. Anti‐inflammatory properties have also been studied by using the carrageenan‐induced paw‐edema model, probably due to the presence of flavonoids and triterpenoids that modulate inflammatory pathways [10, 39]. The cytotoxic effect of *S. samarangense* was evaluated on human cancer cell lines such as lung cancer (A549), breast cancer (MCF‐7), liver cancer (HepG2), and skin cancer in both in vitro and in vivo studies [43, 44, 45, 46]. Both leaf and bark extracts bshowed cytotoxic effects, where bark extract showed a comparatively higher potency owing to the presence of betulinic acid and other bioactive terpenoids [46]. In addition, the topical application of bark extract on animal models significantly enhanced wound healing by accelerating wound contraction and epithelialization due to its antimicrobial and astringent properties [26, 41].

Recent studies indicate that *S. samarangense* leaf extracts possess neuroprotective properties [26, 47]. The antioxidant and anti‐inflammatory activities contribute to the reduction of oxidative stress in neurons, prevent the formation of β‐amyloid plaques, and reduce neuroinflammation, thus helping to protect against cognitive decline [39, 40]. Moreover, its hepatoprotective effect has been demonstrated in liver damage models where its bioactive components reduce hepatotoxicity, normalize liver enzymes (ALT and AST), and improve liver histopathological status [33, 34, 48]. Its fruit's mostly rich flavonoid and polyphenol content has been shown to be beneficial in lowering cholesterol, controlling blood pressure, and preventing lipid peroxidation, which in turn reduces the risk of cardiovascular diseases [40, 49]. The broad spectrum of pharmacological activities of *S. samarangense* showed its significance as a source of natural therapeutics and in traditional and modern pharmacological applications, and the current research focused on a more profound understanding of the molecular mechanisms underlying its bioactivity.

### 3.3 Pharmacologically Important Syzygium Species Versus *S. samarangense*

The genus *Syzygium* consists of more than 1200 species, the majority of which are well characterized pharmacologically. Among all, *Syzygium cumini* (jamun) is the most clinically advanced species for metabolic diseases like diabetes. Its seed and leaf extracts are well known in Ayurveda, and recent studies identified gallic acid and kaempferol as its key phytoconstituents with α‐glucosidase‐inhibitory potency (IC_50_ 0.37 and 0.87 µM) higher than acarbose (5.26 µM) [50]. Conversely, the antidiabetic evidence of *S. samarangense* is limited to crude‐extract animal studies, and no one antidiabetic phytochemical has been validated so far as *S. cumini* [40]. Another genus with clinical benchmarks for antimicrobial application is *Syzygium aromaticum* (clove). It essentially contains eugenol (85%–95% of the total oil composition), an FDA‐recognized topical dental analgesic/antiseptic with antibiofilm potency against multidrug‐resistant ESKAPE (*Enterococcus faecium*, *Staphylococcus aureus*, *Klebsiella pneumoniae*, *Acinetobacter baumannii*, *Pseudomonas aeruginosa*, and *Enterobacter* species) pathogens via a membrane‐perturbation mechanism [51, 52]. However, the antimicrobial potency of *S. samarangense* essential oils (EOs) could not be directly compared to the single‐dominant‐metabolite profile of clove (eugenol) due to the lack of benchmarking against clinical MDR isolates. However, *S. cumini* has a more restricted flavonol‐glycoside profile than *S. samarangense*.

The structural diversity is in line with genus‐wide comparative reviews that indicate *Syzygium aqueum*, *S. aromaticum*, *S. cumini*, and *S. samarangense* as the most phytochemically rich members of the genus overall [30, 53, 54]. Mainly, leaves and fruits of *S. samarangense* contain a broad spectrum of flavonol‐glycoside classes (myricitrin and guaijaverin), acylphloroglucinols (samarones A‐D), chalcones, triterpenoids (betulinic, oleanolic, and asiatic acids), and a novel δ‐lactone (syzysamalactone) that collectively support a broader, multitarget pharmacological footprint spanning antidiabetic, antimicrobial, anticancer, hepatoprotective, and neuroprotective domains [47, 55, 56, 57]. Overall, each genus is dominant with some specific phytochemicals and has reported distinct biological activities; it needs more studies on individual phytochemicals in regional and variety prospective to justify its wide translational values for sustainable drug development.

## 4 Phytopharamacy and MoleculariInsights of *S. samarangense*

The different plant parts and extracts of *S. samarangense* have a wide range of health and therapeutic benefits recorded from various traditional and experimental studies, including blood sugar reduction, antimicrobial activity, hepatoprotective activity, anti‐inflammatory activity, anticancer activity, and wound healing activity. The findings point out the importance of exploring the abundant pool of bioactive molecules in plants that contribute to their biological activity. Researchers have analyzed several parts of *S. samarangense* extracts by gas chromatography–mass spectrometry (GC‐MS), liquid chromatography–mass spectrometry (LC‐MS), and column chromatography and identified many bioactive compounds. Now they are interested more in whether these bioactive work and if they could be used in a nutraceutical or pharmaceutical regimen to treat a specific disorder or disease. Furthermore, an updated list of phytochemicals has been recorded through an extensive literature search (Tables 1, 2, 3, 4). This would be useful to increase our knowledge and help in future drug discovery. We have documented phytochemicals extracted from different plant parts, their solvent system, chemical structure and formula, and their biological activities.

**TABLE 1:** Flavonoids and related polyphenols isolated from the leaves of *S. samarangense* (Blume) Merr. & L.M. Perry leaves.

Sl. No.	Phytochemical	Molecular formula	Solvent system	Plant part	References
1	Kaempferol	C_15_H_10_O_6_	Dichloromethane	Leaves	[86]
2	Myricetin	C_15_H_10_O_8_	Methanol	Leaves	[69]
3	Quercetin	C_15_H_10_O_7_	Methanol	Leaves	[56]
4	3,5‐Di‐*O*‐Methyl gossypetin	C_17_H_14_O_8_	Methanol	Leaves	[69]
5	Myricitrin	C_21_H_20_O_12_	Acetone; methanol; dichloromethane;	Leaves	[56, 71, 86]
6	Myricetin 3‐*O*‐(2′‐*O*‐galloyl)‐α‐rhamnopyranoside	C_28_H_24_O_16_	Acetone	Leaves	[71]
7	Desmanthin‐1	C_28_H_24_O_16_	Dichloromethane	Leaves	[86]
8	Mearnsitrin	C_22_H_22_O_12_	Dichloromethane; ethanol	Leaves	[59, 86]
9	2′‐C‐Methyl‐5′‐*O*‐galloylmyricetin‐3‐*O*‐α‐L‐rhamnopyranoside	C_29_H_26_O_16_	Ethanol	Leaves	[59]
10	Myricitrin 2″‐*O*‐sulfate	C_21_H_19_O_15_Na_2_S	Methanol	Leaves	[57]
11	Quercitrin	C_21_H_20_O_11_	Methanol	Leaves	[56]
12	Hyperin	C_21_H_20_O_12_	Methanol	Leaves	[56]
13	Guaijaverin	C_20_H_18_O_11_	Methanol	Leaves	[56]
14	Reynoutrin	C_20_H_18_O_11_	Methanol	Leaves	[56]
15	Kaempferol‐7‐rhamnoside‐4′‐glucoside	C_27_H_30_O_15_	Methanol	Leaves	[35]
16	Isoengeletin	C_21_H_22_O_10_	Dichloromethane	Leaves	[86]
17	Pinocembrin (5,7‐dihydroxyflavanone)	C_15_H_12_O_4_	Ethanol; methanol	Leaves	[86, 87]
18	(−)‐Strobopinin	C_16_H_14_O_4_	Acetone; dichloromethane	Leaves	[71, 86]
19	8‐Methylpinocembrin	C_16_H_14_O_4_	Dichloromethane	Leaves	[86]
20	Cryptostrobin	C_16_H_14_O_4_	Methanol	Leaves	[57]
21	5,7‐Dihydroxy‐6‐methylflavanone	C_16_H_14_O_4_	Ethanol	Leaves	[87]
22	Demethoxymatteucinol	C_17_H_16_O_4_	Dichloromethane; methanol	Leaves	[81, 86]
23	5,7‐Dihydroxy‐6,8‐dimethylflavanone	C_17_H_16_O_4_	Ethanol	Leaves	[87]
24	7‐Hydroxy‐5‐methoxy‐6,8‐dimethylflavanone	C_18_H_18_O_4_	Dichloromethane; *n*‐hexane	Leaves	[83, 86]
25	(+)‐6,8‐Di‐C‐methyl pinocembrin‐5‐methyl ether	C_18_H_18_O_4_	Dichloromethane	Leaves	[86]
26	5‐*O*‐Methyl‐4′‐desmethoxymatteucinol	C_18_H_18_O_4_	Methanol	Leaves	[79]
27	Cardamonin	C_16_H_14_O_4_	Methanol	Leaves	[56]
28	Stercurensin	C_17_H_16_O_4_	Methanol	Leaves	[56]
29	2′,4′‐Dihydroxy‐6′‐methoxy‐3′‐methylchalcone	C_17_H_16_O_4_	Methanol; *n*‐hexane	Leaves	[79, 83]
30	2′,4′‐Dihydroxy‐6′‐methoxy‐3′,5′‐dimethylchalcone	C_18_H_18_O_4_	Dichloromethane; methanol; *n*‐hexane	Leaves	[79, 83, 86]
31	4′,6′‐Dihydroxy‐2′‐methoxy‐3′,5′‐dimethylchalcone	C_18_H_18_O_4_	Dichloromethane; methanol	Leaves	[86, 88]
32	2′‐Hydroxy‐4′,6′‐dimethoxy‐3′‐methylchalcone	C_18_H_18_O_4_	*n*‐Hexane	Leaves	[83]
33	Aurentiacin	C_18_H_18_O_4_	*n*‐Hexane	Leaves	[61]
34	Syzygiol	C_18_H_18_O_5_	Dichloromethane	Leaves	[86]
35	Uvangoletin	C_16_H_16_O_4_	Dichloromethane	Leaves	[86]
36	2′,4′‐Dihydroxy‐6′‐methoxy‐3′‐methyldihydrochalcone	C_17_H_18_O_4_	Dichloromethane; *n*‐hexane	Leaves	[83, 86]
37	2′,4′‐Dihydroxy‐6′‐methoxy‐3′,5′‐dimethyldihydrochalcone	C_18_H_20_O_4_	*n*‐Hexane	Leaves	[83]
38	(−)‐Epigallocatechin 3‐*O*‐gallate	C_22_H_18_O_11_	Acetone	Leaves	[71]
39	Samarangenin A	C_37_H_28_O	Acetone	Leaves	[70]
40	Samarangenin B	C_44_H_32_O_22_	Acetone	Leaves	[70]

### 4.1 Analgesic and Anti‐inflammatory Activity With Molecular Insights

The crude methanolic leaf extracts of *S. samarangense* at doses of 100 and 200 mg/kg exhibited a significant pain‐relieving effect [58]. Phenolic compounds such as quercetin, gallic acid, and its various derivatives are found in large amounts in leaf extracts, and betulinic acid from the bark has been shown to have pain‐relieving properties [56, 59]. Terpenoids such as α‐pinene and limonene, together with astringent tannins (Tables 2 and 3), have likewise been identified in leaf extracts and are being studied for their analgesic effects [60]. A hexane extract of the leaves containing 2′‐hydroxy‐4′,6′‐dimethoxy‐3′‐methylchalcone (Table 1) showed anti‐inflammatory activity by targeting the activation of nuclear factor‐kappa B (NF‐κB) in mouse macrophages [61]. Similarly, cycloartenyl stearate, lupenyl stearate, sitosteryl stearate, and 24‐methylenecycloartenyl stearate (Table 2) were reported from leaves using a dichloromethane solvent system [62] and displayed strong analgesic and anti‐inflammatory properties at doses between 6.25 and 12.5 mg/kg [62].

**TABLE 2:** Terpenoids and steroids isolated from the leaves of *S. samarangense* (Blume) Merr. & L.M. Perry leaves.

Sl. No.	Phytochemical	Molecular formula	Solvent system	Plant part	References
1	α‐Thujene	C_10_H_16_	Unspecified	Leaves	[60]
2	γ‐Terpinene	C_10_H_16_	Unspecified	Leaves	[60]
3	Terpinen‐4‐ol	C_10_H_18_O	Unspecified	Leaves	[60]
4	Cuminyl aldehyde	C_10_H_12_O	Ethanol	Leaves	[42]
5	Amorphene	C_15_H_24_	Ethanol	Leaves	[42]
6	α‐Copaene	C_15_H_24_	Ethanol	Leaves	[42]
7	α‐Humulene	C_15_H_24_	Ethanol	Leaves	[42]
8	α‐Selinene	C_15_H_24_	Ethanol	Leaves	[42]
9	β‐Selinene	C_15_H_24_	Unspecified	Leaves	[60]
10	β‐Cadinene	C_15_H_24_	Ethanol	Leaves	[42]
11	δ‐Cadinene	C_15_H_24_	Ethanol	Leaves	[42]
12	β‐Caryophyllene	C_15_H_24_	Unspecified	Leaves	[60]
13	Germacrene D	C_15_H_24_	Ethanol	Leaves	[42]
14	τ‐Muurolene	C_15_H_24_	Ethanol	Leaves	[42]
15	β‐Gurjunene	C_15_H_24_	Unspecified	Leaves	[60]
16	Caryophyllene oxide	C_15_H_24_O	Ethanol	Leaves	[42]
17	Spathulenol	C_15_H_24_O	Ethanol	Leaves	[42]
18	α‐Santalol	C_15_H_24_O	Ethanol	Leaves	[42]
19	τ‐Cadinol	C_15_H_26_O	Ethanol	Leaves	[42]
20	τ‐Muurolol	C_15_H_26_O	Ethanol	Leaves	[42]
21	Lupeol	C_30_H_50_O	Dichloromethane; ethanol	Leaves	[86, 87]
22	Betulin	C_30_H_50_O_2_	Ethanol	Leaves	[87]
23	Betulinic acid	C_30_H_48_O_3_	Ethanol	Leaves	[87]
24	*epi*‐Betulinic acid	C_30_H_48_O_3_	Dichloromethane; methanol	Leaves	[83, 86]
25	Betulinic aldehyde	C_30_H_48_O_2_	Ethanol	Leaves	[87]
26	Methyl 3‐*epi*‐betulinate	C_31_H_50_O_3_	Methanol	Leaves	[88]
27	Lupenyl stearate	C_47_H_82_O_2_	Dichloromethane	Leaves	[86]
28	Ursolic acid	C_30_H_48_O_3_	Methanol	Leaves	[88]
29	Ursolic aldehyde	C_30_H_48_O_2_	Ethanol	Leaves	[87]
30	Hydroxyursolic acid	C_30_H_48_O	Dichloromethane	Leaves	[73]
31	Jacoumaric acid	C_39_H_54_O_6_	Methanol	Leaves	[88]
32	Arjunolic acid	C_30_H_48_O_5_	Methanol	Leaves	[88]
33	Methyl 2α,3β,23‐trihydroxy olean‐12‐en‐28‐formate	C_31_H_50_O_5_	Methanol	Leaves	[89]
34	Cycloartenyl stearate	C_18_H_35_O	Dichloromethane	Leaves	[62]
35	24‐Methylenecycloartanyl stearate	C_48_H_86_O_2_	Dichloromethane	Leaves	[62]
36	β‐Sitosterol	C_29_H_50_O	Dichloromethane; ethanol; *n*‐hexane	Leaves	[83, 86, 87]
37	β‐Sitosterol‐D‐glucoside	C_35_H_60_O_6_	Dichloromethane; *n*‐hexane	Leaves	[83, 86]
38	Sitosteryl stearate	C_47_H_84_O_2_	Dichloromethane	Leaves	[62]
39	Stigmasterol‐3‐*O*‐β‐D‐glucoside	C_35_H_58_O_6_	Dichloromethane	Leaves	[86]
40	Campesterol glucoside	C_34_H_58_O_6_	Dichloromethane	Leaves	[86]
41	α‐Carotene	C_40_H_56_	*n*‐Hexane	Leaves	[83]
42	β‐Carotene	C_40_H_56_	*n*‐Hexane	Leaves	[83]
43	Actinidioionoside	C_19_H_34_O_9_	Methanol	Leaves	[57]
44	Actinidioionoside 6′‐*O*‐gallate	C_26_H_38_O_13_	Methanol	Leaves	[57]
45	(3*S*,5*R*,6*R*,7*E*,9*S*)‐3,5,6,9‐Tetrahydroxymegastigman‐7‐ene	C_13_H_24_O_4_	Methanol	Leaves	[57]

**TABLE 3:** Phenolic acids, tannins and other phenolic constituents isolated from the leaves of *S. samarangense* (Blume) Merr. & L.M. Perry leaves.

Sl. No.	Phytochemical	Molecular formula	Solvent system	Plant part	References
1	Gallic acid	C_7_H_6_O_5_	Dichloromethane; methanol	Leaves	[56, 86]
2	Ellagic acid	C_14_H_6_O_8_	Methanol	Leaves	[56]
3	*p*‐Hydroxybenzaldehyde	C_7_H_6_O_2_	Ethanol	Leaves	[87]
4	Methyl salicylate	C_8_H_8_O_3_	Dichloromethane	Leaves	[86]
5	1‐Feruloyl‐β‐D‐glucopyranoside	C_16_H_20_O_9_	Methanol	Leaves	[57]
6	Myrciaphenone A	C_14_H_18_O_9_	Methanol	Leaves	[57]
7	Eugenol	C_10_H_12_O_2_	Dichloromethane	Leaves	[86]
8	Eugenol acetate	C_12_H_14_O_3_	Dichloromethane	Leaves	[86]
9	Anethole	C_10_H_12_O	Ethanol	Leaves	[42]
10	Strictinin	C_27_H_22_O_18_	Dichloromethane	Leaves	[86]
11	Castalagin	C_41_H_26_O_26_	Ethanol	Leaves	[81]
12	Vescalagin	C_41_H_26_O_26_	Ethanol	Leaves	[81]
13	Casuarinin	C_41_H_28_O_26_	Methanol	Leaves	[35]
14	Syzyginin B	C_33_H_24_O_21_	Methanol	Leaves	[35]
15	Eugenin	C_11_H_10_O_4_	Dichloromethane	Leaves	[86]
16	Biflorin	C_16_H_18_O_9_	Dichloromethane	Leaves	[86]
17	2‐Pentadecyl‐5,7‐dihydroxychromone	C_24_H_36_O_4_	Ethanol	Leaves	[55]
18	Jambone E	C_26_H_34_O_4_	Ethanol	Leaves	[55]
19	Jambone F	C_27_H_36_O_4_	Ethanol	Leaves	[55]
20	Jambone G	C_26_H_36_O_4_	Ethanol	Leaves	[55]
21	Jamunone B	C_28_H_40_O_5_	Ethanol	Leaves	[55]
22	Samarone A	C_28_H_43_O_4_	Ethanol	Leaves	[55]
23	Samarone B	C_28_H_41_O_4_	Ethanol	Leaves	[55]
24	Samarone C	C_27_H_39_O_4_	Ethanol	Leaves	[55]
25	Samarone D	C_28_H_39_O_4_	Ethanol	Leaves	[55]
26	5‐Pentadecylresorcinol	C_21_H_36_O_2_	Methanol	Leaves	[90]
27	5‐[(*Z*)‐Pentadec‐10‐enyl]resorcinol	C_21_H_34_O_2_	Methanol	Leaves	[90]
28	5‐Heptadecylresorcinol	C_23_H_40_O_2_	Methanol	Leaves	[90]
29	5‐[(*Z*)‐Heptadec‐10‐enyl]resorcinol	C_23_H_38_O_2_	Methanol	Leaves	[90]
30	5‐[(9*Z*,12*Z*)‐Heptadeca‐9,12‐dienyl]resorcinol	C_23_H_36_O_2_	Methanol	Leaves	[90]
31	5‐[(8*Z*,11*Z*,14*E*)‐Heptadeca‐8,11,14‐trienyl]resorcinol	C_23_H_34_O_2_	Methanol	Leaves	[90]
32	5‐[(*Z*)‐Nonadec‐14‐enyl]resorcinol	C_25_H_42_O_2_	Methanol	Leaves	[90]
33	5‐[(8*Z*,11*Z*,14*Z*)‐Nonadeca‐8,11,14‐trienyl]resorcinol	C_25_H_38_O_2_	Methanol	Leaves	[90]

From a molecular insights point of view, the analgesic and anti‐inflammatory activity of the crude extracts suppresses the NF‐κB axis synergistically rather than on a single compound: the leaf hexane fraction inhibits NF‐κB activation in mouse macrophages [61], and at the whole‐extract level, leaf extract down‐regulates NF‐κB p65 nuclear translocation and its downstream inflammatory signaling while concurrently stimulating endogenous antioxidant defence in an indomethacin‐induced gastropathy model [35]. The overall efficacy is attributable to the combined action of bioactive constituents such as myricetin, syzyginin B, kaempferol‐7‐rhamnoside‐4′‐glucoside, strobopinin, epigallocatechin gallate, casuarinin, and aurentiacin (Tables 1 and 3) acting on this shared pathway, but the exact molecular targets and downstream pathways must be defined before clinical translation can be considered [35, 61].

### 4.2 Antioxidant and Immunomodulatory Potency With Molecular Insights

The methanolic extract of *S. samarangense* leaf, pulp, and seeds possessed higher antioxidant activity [56, 63, 64, 65, 66, 67, 68]. From the seeds, reynoutrin, hyperin, myricitrin, quercitrin, quercetin, and guaijaverin (Table 1), together with gallic acid and ellagic acid (Table 3), were identified in the methanol solvent system [56]. From the pulp, stercurensin and cardamonin (Table 1) and gallic acid and ellagic acid (Table 3) were found, and most of them exhibited higher antioxidant activity in the DPPH assay [56]. Significant antioxidant activity has also been reported for taxiphyllin 6′‐*O*‐gallate (Table 4), myricitrin 2″‐*O*‐sulfate, guaijaverin, myricetin 4′‐methyl ether 3‐*O*‐α‐L‐rhamnopyranoside and myricitrin (Table 1), and gallic acid (Table 3) [57]. Myricetin‐3‐*O*‐α‐rhamnoside and 7,8,3′,4′‐tetrahydroxy‐3,5‐dimethoxyflavone (3,5‐di‐*O*‐methyl gossypetin), both listed in Table 1, have been isolated from the leaves and exhibit potent antioxidant and hepatoprotective effects [69]. The isolated 5,7‐dihydroxy‐6,8‐dimethylflavanone moiety, together with related phenolic and proanthocyanidin metabolites from the ethyl acetate leaf extract, showed the highest antioxidant activities [45, 70]. Mechanistically, these polyphenols act principally as indirect antioxidants that engage the Keap1‐nuclear factor erythroid 2‐related factor 2 (Nrf2) cytoprotective transcriptional program rather than scavenging radicals directly: myricitrin and 3,5‐di‐*O*‐methyl gossypetin up‐regulate Nrf2 and downstream manganese superoxide dismutase (Mn‐SOD‐3) in keratinocytes [69], while the aqueous leaf extract independently boosts Nrf2‐antioxidant response element (Nrf2‐ARE) luciferase activity and induces the Nrf2 target enzymes hem oxygenase‐1 (HO‐1), NAD(P)H quinone dehydrogenase 1 (NQO1), and UDP‐glucuronosyltransferase 1A (UGT1A) in HepG2‐C8 cells [44].

**TABLE 4:** Miscellaneous constituents (fatty acids, esters, lactones, cyanogenic glycosides, and structurally unassigned compounds) isolated from the leaves of *S. samarangense* (Blume) Merr. & L.M. Perry leaves.

Sl. No.	Phytochemical	Molecular formula	Solvent system	Plant part	References
1	Palmitic acid	C_16_H_32_O_2_	Ethanol	Leaves	[42]
2	Oleic acid	C_18_H_34_O_2_	Ethanol	Leaves	[42]
3	Benzyl benzoate	C_14_H_12_O_2_	Dichloromethane	Leaves	[86]
4	Syzysamalactone	C_10_H_14_O_2_	Unspecified	Leaves	[47]
5	6‐Pentyl‐α‐pyrone	C_10_H_14_O_2_	Unspecified	Leaves	[47]
6	(*R*)‐Prunasin	C_14_H_17_NO_6_	Methanol	Leaves	[57]
7	Taxiphyllin 6′‐*O*‐gallate	C_21_H_21_O_11_N	Methanol	Leaves	[57]
8	Sysamarin A	C_45_H_76_O_6_	Methanol	Leaves	[91]
9	Sysamarin B	C_47_H_80_O_6_	Methanol	Leaves	[91]
10	Sysamarin C	C_49_H_84_O_6_	Methanol	Leaves	[91]
11	Sysamarin D	C_49_H_82_O_6_	Methanol	Leaves	[91]
12	Sysamarin E	C_45_H_76_O_6_	Methanol	Leaves	[91]
13	Sysamarin F	C_47_H_80_O_6_	Methanol	Leaves	[89]
14	Sysamarin G	C_50_H_86_O_6_	Methanol	Leaves	[89]

*S. samarangense* leaf extracts have also been shown to enhance several aspects of the immune response. Around 16 flavonoids were isolated from the acetone extract (Table 1), of which four, such as strobopinin, myricetin 3‐*O*‐(2″‐*O*‐galloyl)‐α‐rhamnopyranoside, (−)‐epigallocatechin 3‐O‐gallate, and myricetin 3‐O‐α‐rhamnopyranoside, demonstrated immunomodulatory effects in a dose‐dependent manner via interleukin‐2 (IL‐2) blockade and interferon‐γ (IFN‐γ) production [71]. A further study isolated 16 flavonoids from the leaves and evaluated their immunomodulatory efficacy using peripheral blood mononuclear cells [71]. Among them, (−)‐strobopinin, myricetin 3‐*O*‐(2″‐*O*‐galloyl)‐α‐rhamnopyranoside, (−)‐epigallocatechin 3‐O‐gallate, and myricetin 3‐*O*‐α‐rhamnopyranoside showed inhibitory potency at IC_50_ values of 36.3, 11.9, 28.9, and 75.6 µM, respectively [71]. The inhibitory mechanism operates through dose‐dependent suppression of IL‐2 and IFN production [71], placing the immunomodulatory action of this flavonoid set on a cytokine‐transcriptional axis that is mechanistically continuous with the NF‐κB suppression. A separate study analyzed antioxidant activity, cell viability, and human primary skin irritation for leaf and branch extracts [72]. Based on IC_50_ values, the branch extract showed comparatively higher antioxidant activity (DPPH: 24.158 ± 0.685 µg/mL; ABTS: 10 µg/mL; DMPD: 53.269 ± 0.386 µg/mL) and phenolic content (76.3820 ± 1.085 mg GAE/g) than the leaf extract (DPPH: 21.352 ± 4.683 µg/mL; ABTS: 10 µg/mL; DMPD: 57.278 ± 1.186 µg/mL; 66.778 ± 1.64 mg GAE/g). Both extracts were found to be safe for application as natural cosmetic materials with 78%–80% viability at 25 µg/mL for HaCaT human keratinocyte cells and negligible skin irritation [72].

### 4.3 Antibacterial and Antifungal Potency With Molecular Insights

From bioassay‐guided fractionation, around ten triterpene‐class phytochemicals such as betulinic acid, oleanolic acid, 2‐hydroxyoleanolic acid, 2‐hydroxyursolic acid, arjunolic acid, asiatic acid, terminolic acid, 6‐hydroxyasiatic acid, arjunolic acid 28‐β‐glucopyranosyl ester, and asiatic acid 28‐β‐glucopyranosyl ester were found in *S. samarangense* leaf (Table 2; Figure 3). Among all, only arjunolic acid and asiatic acid showed significant antibacterial activity against *Escherichia coli*, *Bacillus subtilis*, and *Shigella sonnei* [73]. Ethanolic leaf extracts were effective against *Bacillus cereus* and *Salmonella enterica* at a minimum inhibitory concentration (MIC) of 78 µg/mL but were considerably less effective against *E. coli* and *Enterobacter aerogenes* (MIC 2500 µg/mL) [74]. A set of chalcone, triterpene, and sterol constituents (2′,4′‐dihydroxy‐6′‐methoxy‐3′‐methylchalcone; 2′,4′‐dihydroxy‐6′‐methoxy‐3′,5′‐dimethylchalcone; 2′‐hydroxy‐4′,6′‐dimethoxy‐3′‐methylchalcone (Table 1); squalene, betulin, lupeol, and β‐sitosterol (Table 2) was reported from the dichloromethane leaf extract and exhibited low to moderate antifungal and antibacterial activity [75]. These constituents showed lower activity against *E. coli*, *Staphylococcus aureus*, and *Pseudomonas aeruginosa;* moderate antifungal activity against *Candida albicans;* and were inactive against *B. subtilis* and *Aspergillus niger* [75].

**FIGURE 3:**
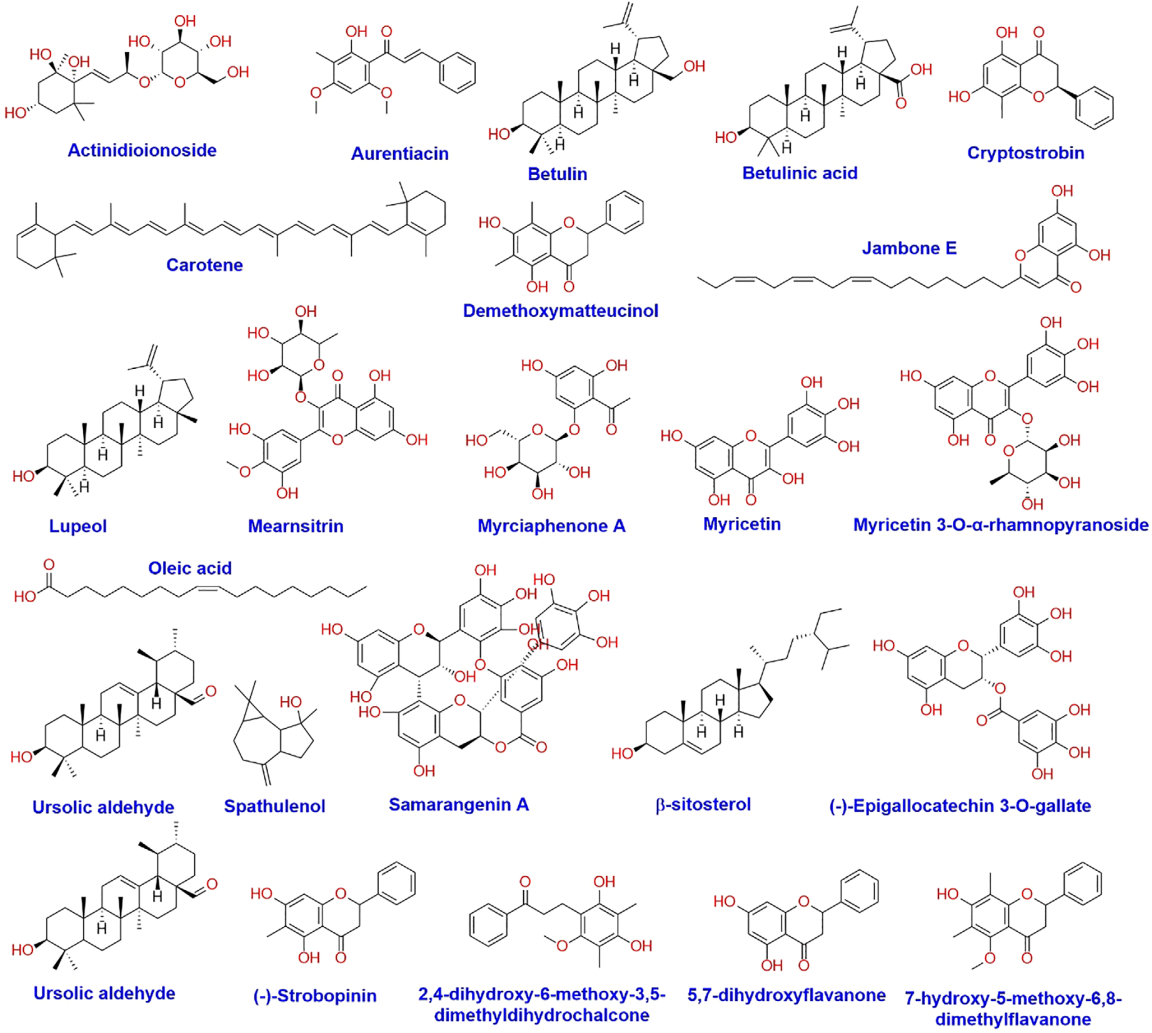
The chemical structure of some of the bioactive phytochemicals isolated from *S. samarangense* leaves. After a literature search, the chemical structure was presented using ChemDraw 20.0 software.

Essential oils (EOs) of *S. samarangense* leaf were found to contain globulol (52.09%) and α‐pinene (30.18%) together with caryophyllene oxide, *p‐*cymene, and γ‐terpinene (8%–20%) (terpenoid constituents, Table 2); these mixtures synergistically exhibited broad‐spectrum antimicrobial potential against *E. coli* (MIC 3.75 µL/mL) and against biofilm‐forming *C. albicans* [41]. A comparable study on leaf EOs reported that hydrodistillation gave higher potency than supercritical fluid extraction, inhibiting *E. coli* growth (MIC 3.75 µL/mL) and suppressing *C. albicans* biofilm formation by 83.43% and 87.27%, respectively; GC‐MS identified globulol (52.09%), γ‐terpinene (36.06%), α‐pinene (30.18%), caryophyllene oxide (8.19%–18.48%), and *p*‐cymene (16.02%–19.50%) [41]. From a molecular mechanism perspective, no such mode of action has yet been established for the antimicrobial action, but the activity trails consistently with the lipophilic triterpene, sterol, and monoterpene fractions (Table 2) rather than with the polar polyphenols, which is consistent with a membrane‐directed, non‐receptor‐mediated mode of action. In addition, this has also not been tested directly for this species, and no target‐level evidence (membrane permeabilization assay, efflux pump, or cell‐wall‐synthesis readout) has been reported; we need to explore taking both extracts and phytoconstituents derived from *S. samarangense*.

Fresh bark extracted with three solvent systems (*n*‐hexane, dichloromethane, and methanol) gave MIC values of 64–512 µg/mL against *S. aureus*, *E. coli*, and *Salmonella typhi*, with an LC_50_ of 30.031 µg/mL in the brine shrimp cytotoxicity model [76]. The ethanol extract showed the strongest effect, with maximum inhibition zones of 22.03 ± 0.096 mm against *E. coli* and 17 ± 0.182 mm against *K. pneumoniae* at 1 mg/disc. Although no such bioactive compounds were isolated, total phenolic and flavonoid contents at 162.03 ± 0.05 to 90.42 ± 0.28 ppm and 148.24 ± 0.07 to 99.64 ± 0.08 ppm indicate the presence of different types of phenolic and flavonoid constituents [77]. In a related study, aqueous and ethanolic fruit extracts were tested against three oral pathogens (*Porphyromonas gingivalis*, *Fusobacterium nucleatum*, and *Aggregatibacter actinomycetemcomitans*): the ethanolic extract was most potent at 0.4 µg/mL and the aqueous extract at 1.6 µg/mL against *F. nucleatum*, with no activity against the other two species [78]. A study on crude leaf extract against six pathogenic fungal strains recorded minimum fungicidal concentrations of 100–125 mg/mL in both in vitro and in vivo experiments [36]; Figure 4.

**FIGURE 4:**
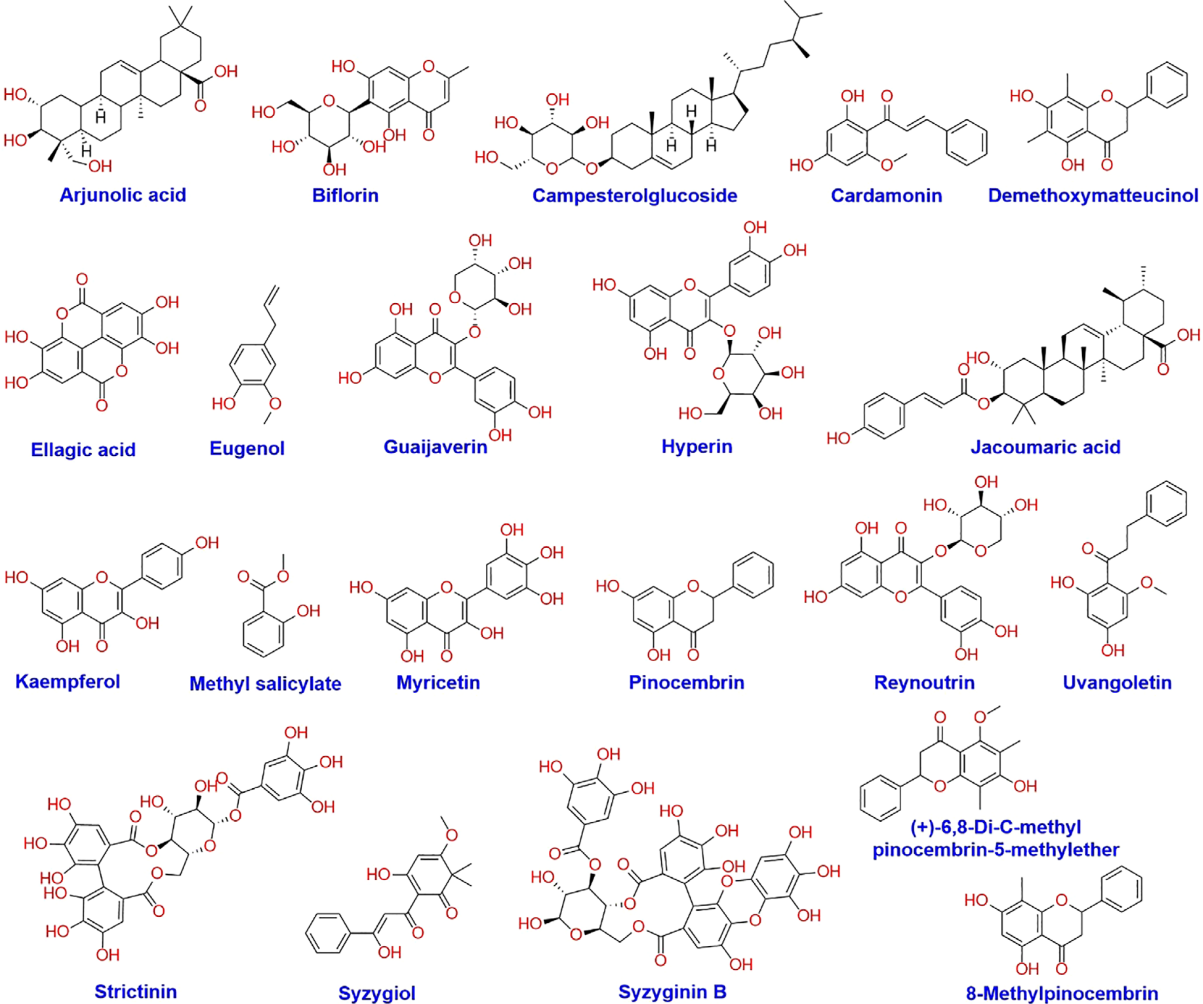
The chemical structure of some of the bioactive phytochemicals isolated from *S. samarangense* fruits and bark. After a literature search, the chemical structure was presented using ChemDraw 20.0 software.

### 4.4 Antidiabetic and Anticancer Activity With Molecular Insights

Three flavonoids from the leaves of *S. samarangense*, namely, 2′,4′‐dihydroxy‐3′,5′‐dimethyl‐6′‐methoxychalcone, 5‐O‐methyl‐4′‐desmethoxymatteucinol, and 2′,4′‐dihydroxy‐6′‐methoxy‐3′‐methylchalcone (Table 1), were isolated and found to lower blood sugar in diabetic mice at 1.0 mg/20 g body weight [79]. Only 2′,4′‐dihydroxy‐3′,5′‐dimethyl‐6′‐methoxychalcone produced a decrease of up to 35% at *t* = 45 min after glucose administration [79]. The methanolic leaf extract was most effective at lowering blood sugar, reducing levels by up to 59.3% in Swiss albino mice at 400 mg/kg body weight, although no bioactive phytochemical was isolated from the crude extract [29]. Fresh fruit juice was investigated against streptozotocin (STZ)‐induced pancreatic β‐cell apoptosis in diabetic rats at 100 mg/kg for 30 days [80]. Leaf extract at 100 mg/kg also improved glycemic parameters (serum glucose, insulin, and lipid peroxide levels) in STZ‐diabetic rats, and the reported phytochemicals were subsequently screened for target‐specific binding against two diabetes‐associated targets by molecular docking [40]. A recent study of antidiabetic constituents of the leaves reported a new anacardic acid derivative, 2‐[(*Z*)‐nonadec‐14‐enyl]‐6‐hydroxybenzoic acid, together with four known congeners (the alkyl‐benzoic acid series structurally related to the alkylresorcinols in Table 3); enzyme assays confirmed significant α‐glucosidase inhibitory efficacy with IC_50_ values of 0.75–3.60 µM [38].

The antidiabetic and anticancer activities of *S. samarangense* converge on a single caspase‐dependent mitochondrial apoptotic axis that is exploited in opposite directions depending on the target tissue: fruit juice down‐regulates cleaved caspase‐3 and Bax while up‐regulating the anti‐apoptotic proteins Bcl‐2 and Bcl‐xl to preserve pancreatic β‐cells, apparently by blocking oxidative stress and proinflammatory cytokine signalling [80], whereas the same caspase‐3/Bax/Bcl‐2 axis is driven toward apoptosis induction by the cytotoxic chalcones and acylphloroglucinols in tumor cells. A second, independent anticancer axis operates at the level of DNA repair, with the ellagitannins castalagin and vescalagin inhibiting poly(ADP‐ribose) polymerase 1 (PARP1) and apparently dual‐targeting DNA topoisomerase II [81]. A number of chalcone‐ and flavonoid‐class compounds were reported from methanolic extracts of fruit pulp and seeds, of which 2′,4′‐dihydroxy‐3′,5′‐dimethyl‐6′‐methoxychalcone, stercurensin, cardamonin, and (*S*)‐pinocembrin (Table 1) showed potent cytotoxicity with IC_50_ values against the human colon cancer SW‐480 line of 10, 25, 35, and 60 µM, respectively [56]. The methanolic pulp extract itself gave an IC_50_ of 60 µM, whereas the methanolic seed extract was not cytotoxic against SW‐480 [56]. The same group reported five flavonoids from the leaves (2′‐hydroxy‐4′,6′‐dimethoxy‐3′‐methylchalcone; 2′,4′‐dihydroxy‐6′‐methoxy‐3′,5′‐dimethylchalcone; 2′,4′‐dihydroxy‐6′‐methoxy‐3′‐methylchalcone; 2′,4′‐dihydroxy‐6′‐methoxy‐3′‐methyldihydrochalcone; and 2′,4′‐dihydroxy‐6′‐methoxy‐3′,5′‐dimethyldihydrochalcone (Table 1) and showed potential cytotoxicity against Chinese hamster ovary (CHO‐AA8) cells and the human mammary adenocarcinoma lines MCF‐7 and SKBR‐3 [56]. Note that among all, the 2′,4′‐dihydroxy‐6′‐methoxy‐3′,5′‐dimethylchalcone showed the most effectiveness against MCF‐7 (0.0015 ± 0.0001 nM) and SKBR‐3 (0.0128 ± 0.0006 nM) in comparison to the rest of the isolated flavonoids [56].

Bioassay‐guided extraction yielded 17 phytochemicals, including *R*‐prunasin and the megastigmane glucosides (Table 4), lupeol, betulinic acid, β‐sitosterol glucoside, and actinidioionoside (Table 2), demethoxymatteucinol, cryptostrobin, guaijaverin, myricetin 4′‐methyl ether 3‐O‐α‐L‐rhamnopyranoside, myricitrin (Table 1), myrciaphenone A, 1‐feruloyl‐β‐D‐glucopyranoside, and gallic acid (Table 3) [57]. In antitumor testing against the human lung cancer A549 line, six candidates, the cyanogenic glucoside (73.9 ± 10.2 µM), the rhamnoside (76.0 ± 11.6 µM), cryptostrobin (75.4 ± 13.5 µM), betulinic acid (56.4 ± 16.8 µM), myricitrin (59.4 ± 10.3 µM), and gallic acid (43.2 ± 23.2 µM), exhibited weak antitumor activity [57]. Screening of plant extracts identified vescalagin and castalagin (Table 3) in the leaves [81], where castalagin was the more potent PARP1 inhibitor (IC_50_ = 0.86 µM) relative to vescalagin (IC_50_ = 2.67 µM). Four acylphloroglucinol derivatives, samarones A–D, together with jambones E–G, jamunone B, and 2‐pentadecyl‐5,7‐dihydroxychromone (Table 3), were isolated from ethanolic leaf extracts [55]. With the exception of samarone D, all were tested for cytotoxicity against HepG2 and MDA‐MB‐231 cells and displayed potent activity (IC_50_ 1.73–32.90 µM against HepG2; 4.02–37.83 µM against MDA‐MB‐231), with greater potency against HepG2 throughout and jambone G as the lead candidate against both lines (1.73 ± 0.66 and 4.02 ± 0.87 µM) [55]. In addition, the skin cancer chemopreventive potential of leaf water extract was examined [44]: the extract boosted Nrf2‐ARE luciferase activity and raised RNA and protein levels of Nrf2 and its downstream enzymes (HO‐1, NQO1, and UGT1A) in HepG2‐C8 cells in a concentration‐dependent manner and shielded mouse epidermal JB6 P+ cells from TPA‐induced carcinogenesis [44].

### 4.5 Hepatoprotective and Neuroprotective Activity With Molecular Insights

Fresh fruit juice of *S. samarangense* was investigated for hepatoprotective potency against alcohol‐induced liver damage [34]. In C57BL/6 mice, alcohol combined with a fruit‐water mixture at 10 mL/kg body weight daily reduced aspartate transaminase, alanine transaminase, total bilirubin, triglyceride, and malondialdehyde levels, indicating protection against alcohol‐induced hepatic damage, but no responsible bioactive compounds were identified from the extracts [34]. A separate study examined the gastroprotective effect of the leaves against indomethacin‐induced (60 mg/kg) injury in a mouse model at 100 and 200 mg/kg for 8 days [35]. This protection is NF‐κB‐dependent and Nrf2‐linked, where the extract decreased NF‐κB p65 expression and downstream inflammatory signaling while stimulating cellular antioxidant defence [35], the same dual engagement described for the antioxidant and anti‐inflammatory activities. Molecular docking of several phytochemicals against prostaglandin receptors EP3 and EP4 indicated potential binding for all candidates, with tellimagrandin II showing the highest potency against EP3 (−31.31 kcal/mol) and casuarinin against EP4 (−25.96 kcal/mol.) (Table 3; for more details, see Section 4.7).

Recently a study reported that peeled fruit (water extraction) contains mannose (2.20), rhamnose (3.94), glucuronic acid (4.45), galacturonic acid (8.56), glucose (8.86), galactose (30.82), arabinose (39.78), and fucose (1.48) in molar ratio [82]. The isolated polysaccharide (Araf‐(1→, Glcp‐(1→, →2)‐Araf‐(1→, →3)‐Galp‐(1→, →3)‐Araf‐(1→ and →6)‐Galp‐(1→) showed significant protective effects on EC‐treated L02 cells by suppressing cytotoxicity and genotoxicity, reducing reactive oxygen species (ROS), and increasing mitochondrial membrane potential (MMP) and glutathione (GSH) levels [82]. The ROS‐suppressing and MMP‐preserving profile of this polysaccharide places it on the same mitochondrial‐protection axis described for β‐cell preservation in Section 4.4, indicating that the hepatoprotective and antidiabetic effects of this species are unlikely to be mechanistically independent. The polysaccharide is therefore a plausible therapeutic agent or supplement for hepatic oxidative damage [82].

The neuroprotective effects of the methanolic leaf extract were investigated in mice at 100 and 200 mg/kg body weight, and central nervous system (CNS) depressant activity was evaluated by the reduction of locomotor and exploratory activity in open field and hole cross tests [58]. The extract showed strong analgesic and mild anti‐inflammatory effects alongside significant CNS activity, supporting the traditional use of this plant for inflammatory pain relief [58]. From the hexane leaf extract, α‐carotene, β‐carotene, lupeol, betulin, *epi*‐betulinic acid, β‐sitosterol, and β‐D‐sitosteryl glucoside (Table 2) were isolated together with 2′,4′‐dihydroxy‐6′‐methoxy‐3′‐methylchalcone, 2′‐hydroxy‐4′,6′‐dimethoxy‐3′‐methylchalcone, 2′,4′‐dihydroxy‐6′‐methoxy‐3′,5′‐dimethylchalcone, 2′,4′‐dihydroxy‐6′‐methoxy‐3′‐methyldihydro ‐chalcone, 2′,4′‐dihydroxy‐6′‐methoxy‐3′,5′‐dimethyldihydrochalcone, and 7‐hydroxy‐5‐methoxy‐6,8‐dimethylflavanone (Table 1). Here the mechanism is distinct from the transcriptional axes above and is instead enzyme‐inhibitory: *epi*‐betulinic acid, 2′,4′‐dihydroxy‐6′‐methoxy‐3′‐methyldihydrochalcone, and 7‐hydroxy‐5‐methoxy‐6,8‐dimethyl flavanone act as thrombin and prolyl endopeptidase inhibitors, giving neuroprotective efficacy in the range 12–15 µM [83]. From ripe fresh fruits, a new δ‐lactone derivative, syzysamalactone, was isolated together with the previously reported 6‐pentyl‐α‐pyrone Table 4; [47]; assessed in the SH‐SY5Y human neuroblastoma line, syzysamalactone showed the greater neuroprotective effect (EC_50_ 0.29 ± 0.03 µM) relative to 6‐pentyl‐α‐pyrone (1.28 ± 0.06 µM) [47]. Overall, the various plant parts of *S. samarangense* yield numerous potent phytochemicals contributing to CNS depression by mitigating neuroinflammation and oxidative stress. Several additional phytochemicals listed in Tables 1, 2, 3, 4 remain biologically uncharacterized and should be evaluated for future therapeutic use [84, 85].

### 4.6 Integrative Mechanistic Cross‐Talk Pathways and Computational Pharmacology

The individual mechanistic findings are presented, where three axes recur across the *S. samarangense* literature: Nrf2‐mediated cytoprotection, NF‐κB‐mediated anti‐inflammation, and caspase/PARP1‐mediated apoptotic modulation. Because Nrf2 and NF‐κB are known to cross‐talk antagonistically, Nrf2 activation typically dampens NF‐κB‐driven transcription of pro‐inflammatory cytokines. The first two axes are likely mechanistically linked rather than independent. This cross‐talk has not yet been tested directly for *S. samarangense* and represents a concrete, testable hypothesis for future work. Similarly, the PARP1 inhibition displayed by castalagin and vescalagin is consistent with the synthetic‐lethality strategies used clinically for BRCA‐deficient tumors; this parallel has not previously been drawn for *S. samarangense* metabolites and warrants direct comparison with clinical PARP inhibitors such as olaparib. Framing the evidence around these three interacting axes, rather than as a list of activities, is intended to differentiate this review from earlier, purely descriptive reports on the species.

In line with the recommendation to separate computational predictions from wet‐laboratory evidence, the in silico investigations reported for *S. samarangense* are consolidated here. A molecular docking of reported leaf phytoconstituents against two diabetes‐associated targets, peroxisome proliferator‐activated receptor gamma (PPARγ) and glucagon‐like peptide‐1 receptor (GLP‐1R), and paired these predictions with in vivo confirmation in streptozotocin‐diabetic rats [40]. Similarly, some leaf‐derived tannins (Table 3) against prostaglandin E receptors EP3 and EP4, where tellimagrandin II (−31.31 kcal/mol against EP3) and casuarinin (−25.96 kcal/mol against EP4) were the best candidates, to support the NF‐κB‐mediated gastroprotective effect [35]. However, neither study involved re‐docking validation against a co‐crystallized reference ligand, grid‐box parameters, or molecular dynamics/molecular mechanics Poisson–Boltzmann surface area follow‐up of the top hits. Therefore, the computational evidence for *S. samarangense* should be considered hypothesis‐generating and target‐prioritizing, rather than confirmatory of the binding mechanism or mode of action. Future in silico work on this species should be held to a higher level of reporting standards to achieve parity with the computational pharmacology work available for related *Syzygium* species.

## 5 Clinical Relevance, Translational Potential, and Safety Considerations

The above sections have shown that *S. samarangense* is a preclinical active species with a rich chemistry [32, 47, 53, 55, 56, 57, 59, 60, 61, 62, 69, 70, 71, 73, 76, 77, 78, 79, 80, 81, 82, 83, 84, 86, 87, 88, 89, 90, 91, 92, 93]. A crucial element in the evaluation of this section is to clearly distinguish where the evidence ends, since most of the ethnopharmacological efforts fail at the interface between a documented phytochemical inventory and a therapeutic agent [1, 2, 3, 4, 13, 14, 15]. The section outlines the clinical limitations of *S. samarangense*, aiming to motivate future researchers to pursue essential research in this area.

### 5.1 Lack of Human Evidence With Pharmacokinetics, Bioavailability, and Delivery

A structured check of clinical trial registries (ClinicalTrials.gov [94]; WHO ICTRP [95]) and of the published literature did not identify any completed or ongoing registered human trial evaluating *S. samarangense* extracts or isolated phytoconstituents. This finding sharply contrasts with *S. cumini*, whose seed and leaf preparations are already included in marketed Ayurvedic antidiabetic formulations [50], as well as with clove and its principal constituent eugenol, which have established over‐the‐counter dental and topical antiseptic uses [51, 52]. All efficacy data summarized in Sections 3 and 4 derive exclusively from in vitro assays [55, 56, 57, 71, 81], rodent models [29, 35, 58, 79, 80], and a small number of in silico predictions [40, 96]. Consequently, the statement of chemical and preclinical promise of *S. samarangense* needs more clinical work to be focused on utilizing the natural resources in mainstream drug discovery and perfecting a plan for a gateway to future drug development with stronger clinical evidence.

On the other hand, pharmacokinetic characterization is comparably underdeveloped: none of the studies reviewed oral bioavailability, plasma half‐life, protein binding, metabolite profiling, or tissue distribution for any *S. samarangense* phytoconstituent in vivo [29, 34, 35, 58, 79, 80]. Several of the most active reported metabolites are glycosylated flavonoids, namely, myricitrin, guaijaverin, and quercitrin, generally associated with poor intestinal absorption and extensive first‐pass, gut‐microbiota‐mediated deglycosylation [56, 57, 69]. Reported in vitro IC_50_ values for these compounds may therefore considerably overestimate achievable in vivo potency [57, 97, 98]. The problem compounds the mechanistic argument developed in the above sections, which are all intracellular, so any claim that a given metabolite engages them in vivo presupposes systemic and intracellular exposure that has never been measured for this species. Therefore, active formulation strategies, including nanocarrier‐based delivery systems and targeted chemical modification, can improve the stability and absorption of polyphenolic constituents and have been applied successfully to structurally comparable flavonoids [99, 100]. These approaches should be understood as a response to a characterized pharmacokinetic deficiency.

### 5.2 Toxicology and Translation Dose Validation for Mainstream Application

Toxicological data is another translational limitation with a lack of acute (OECD 423) or repeated‐dose sub‐chronic (OECD 408) including genotoxicity or reproductive and developmental toxicity assessment [35, 58, 80]. Long‐term safety, dose‐response relationships in humans, and drug–drug interaction potential all remain uncharacterized. Therefore, there is a need for more clinical research attention on cytochrome P450 modulation of the phenolic and flavonoid class of constituents, since flavonoids and hydrolysable tannins are established modulators of CYP3A4 and CYP2C9 activity [101, 102]. This is clinically consequential in precisely the settings where the plant is most likely to be used, in which herbal preparations are frequently taken alongside conventional pharmacotherapy without disclosure to a prescriber [13, 14, 15]. Reported efficacious doses span 6.25–400 mg/kg body weight depending on extract and model [29, 58, 62, 79] and are not readily convertible to a human‐equivalent dose in the absence of a standardized extract with defined marker‐compound content; body‐surface‐area‐based allometric conversion presupposes a chemically defined test article [103].

### 5.3 Standardization, Quality Control, and Regulatory Barriers

Standardization remains the most immediate practical obstacle. Phytochemical composition varies intrinsically with genotype, growing environment, harvest stage, and postharvest processing, which undermines batch‐to‐batch consistency and makes bioequivalence difficult to demonstrate [22, 30, 46, 50]. The metabolite profile recovered from *S. samarangense* leaf is strongly solvent‐dependent, with acetone [70, 71], dichloromethane [62, 86], ethanol [42, 55, 87], methanol [56, 57, 88, 89], and *n*‐hexane [83] each yielding a partially distinct set of constituents (Tables 1, 2, 3, 4). An extract specification for this species must therefore define the solvent system as well as the plant part and marker content, since ‘leaf extract’ alone does not identify a reproducible material. Validated HPLC and LC‐MS methods quantifying selected markers, myricitrin and guaijaverin, are reasonable candidates on grounds of abundance and reported activity [56, 57, 69] and are a prerequisite for any subsequent step [103]. The regulatory environment for herbal medicines compounds this difficulty, varying substantially between jurisdictions in its evidentiary requirements and creating uneven barriers to market approval [13, 14, 15, 104]. Addressing these issues requires interdisciplinary cooperation between ethnobotanists, phytochemists, pharmacologists, and clinical researchers, supported by robust compound and activity databases and by collaborative research networks [1, 2, 3, 4]. Given that plant‐based preparations remain widely used as affordable alternatives in low‐ and middle‐income economies [13, 14, 15], resolving quality control is a question of patient safety in current practice, not only of future licensing.

Simultaneously, any scale‐up of *S. samarangense* for pharmaceutical use raises supply and equity questions that are best settled before demand exists rather than after. Effective agricultural and collection practices, together with in situ and ex situ conservation measures, would limit the risk of overexploitation and ecological degradation associated with wild collection of medicinal species [13, 14, 15, 105]. Equally, where the therapeutic lead derives from documented traditional use, equitable benefit‐sharing with the source and indigenous communities is both an ethical obligation and, under the Nagoya Protocol on access and benefit‐sharing, an increasingly enforceable legal one [1, 2, 3, 4, 106].

### 5.4 A Translational Roadmap and Future Remarks on *S. samarangense*

For *S. samarangense* to progress credibly toward drug development, we recommend the following steps and investigation reports for translation success, such as chemical standardization in respect to solvent system, preclinical toxicology conducted to OECD guidelines (acute and repeated‐dose sub‐chronic), including genotoxicity and reproductive/developmental toxicity testing, pharmacokinetic profiling of lead compounds covering absorption, distribution, metabolism, and elimination, with explicit attention to gut‐microbiota‐mediated deglycosylation of the flavonoid glycosides and evaluation of cytochrome P450 interaction potential, mechanistic confirmation of the Nrf2, NF‐Κb, and caspase/PARP1 pathways using pathway‐specific inhibitors or knockdown and knockout models rather than correlative expression data alone, and last but not least, investigator‐initiated Phase 0/I human studies addressing safety and pharmacokinetics as a prerequisite for any efficacy claim [44, 79, 102, 103, 104, 105, 106, 107]. In addition, contemporary discovery methods such as high‐throughput screening, combinatorial chemistry, bioinformatics, and network‐pharmacology approaches also guided toward understanding the drug‐ability information and moving with prioritized leads for high clinical success [46, 96, 108].

*S. samarangense* presents an unusually broad phytochemical class, such as flavonoids, chalcones, triterpenoids, phytosterols, tannins, and phenolic acids (Tables 1, 2, 3, 4), and accordingly showed a broad preclinical activity profile [47, 54, 55, 56, 57, 58, 80, 81, 90], and its constituents converge on a small number of well‐characterized and druggable signaling axes [35, 44, 61, 69, 80, 81] rather than acting through unrelated mechanisms. That convergence is the strongest argument for continued investment in the species. It is not, however, a substitute for the evidence that does not yet exist [94, 95]. Positioning *S. samarangense* alongside *S. cumini* [50] and *S. aromaticum* [51, 52] as a genus member of demonstrated rather than projected translational value will require completion of the stages set out above, and particularly the pharmacokinetic [97, 98] and toxicological [105, 107] work that no published study has yet undertaken. Framed with that qualification, the species remains a genuine and tractable bridge between documented traditional use and modern drug discovery [1, 2, 3, 4].

## 6 Conclusion

The wide range of phytochemicals and biological records can be used as an alternative medicine for the popular edible nutritional plant *S. samarangense*. It is rich in bioactive flavonoids, tannins, terpenoids, saponins, and phenolic compounds, which provide it several health benefits against diabetes, bacteria, inflammation, and cancer. Experimental studies have demonstrated the potential of *S. samarangense* to modulate several important molecular pathways involved in metabolic disorders, infectious diseases, and cancer progression. The exploration of the full pharmaceutical potential and the overcoming of the pharmacological limitations require advanced omics technologies and in vivo studies in collaboration with ethnobotanists, pharmacologists, and clinicians. In conclusion, the new ethnomedicinal and scientific information about *S. samarangense* provides us a favorable chance to make new natural and cheap health care products.

## Author Contributions

**Sujit Kumar Patro**: data collection, writing, review and editing. **Shasank S. Swain**: conceptualization, original draft, visualization, review and editing; **Rudra Narayan Sahoo**: formal analyses, review and editing. **Ashirbad Nanda**: conceptualization, supervision, review and editing.

## Conflicts of Interest

The authors declare no conflicts of interest.

## Data Availability

The data that support the findings of this study are available from the corresponding author upon reasonable request.
